# Evaluation of Stability of Rotating Hinge Knee Prostheses: A Biomechanical Study

**DOI:** 10.1155/2013/701693

**Published:** 2013-07-18

**Authors:** Joerg Friesenbichler, Andreas Leithner, Mathias Glehr, Patrick Sadoghi, Werner Maurer-Ertl, Alexander Avian, Reinhard Windhager

**Affiliations:** ^1^Department of Orthopedic Surgery, Medical University of Graz, Auenbruggerplatz 5, 8036 Graz, Austria; ^2^Institute for Medical Informatics, Statistics and Documentation, Medical University of Graz, Auenbruggerplatz 2, 8036 Graz, Austria; ^3^Department of Orthopedic Surgery, Medical University of Vienna, Waehringer Gürtel 18-20, 1090 Vienna, Austria

## Abstract

*Purpose*. Rotating hinge knee prostheses should provide a stable situation following reconstruction. We performed a biomechanical analysis to establish the association between design of the central rotational stem (peg) and implant's stability, in a theoretical setting. *Methods*. Six different rotating hinge designs were tested, and three observers performed two different measurements with a custom made biomechanical apparatus and laterally directed pressure. The aim was to assign the degree of tilting of the peg within the vertical post-in channel by extending the distraction as well as the maximum amount of distraction before the peg's dislocation. An intraclass-correlation coefficient (ICC) was calculated to determine the observer's reliability. *Results*. Implant designs with cylindrical pegs of different lengths were superior to implant designs with conical or other shaped pegs concerning stability and maximum amount of distraction before dislocation, showing steep rising distraction-angular displacement curves. The ICC at 15 mm and 25 mm of distraction revealed high interobserver reliability (*P* < 0.001). *Conclusion*. The biomechanical analysis showed that rotating hinge prostheses with long and cylindrical pegs have the highest stability at any given amount of distraction. Designs with shorter and markedly tapered pegs may become unstable under conditions of mild joint distraction which has to be proven in future in vivo investigations.

## 1. Introduction

Limb salvage surgery and revision arthroplasty are more demanding procedures than primary total arthroplasty. Components of these procedures are a stable fixation of the prostheses to the host bone, joint line restoration, and a stable range of motion consistent with the patient's daily activities. These goals should be accomplished with the least degree of prosthetic constraint possible. Therefore, implant selection should be based on the amount of bone loss, the status of ligaments, and the soft-tissue stabilizing structures [1–5]. In cases of global insufficiency or complete loss of all joint ligaments the increasing constraint from a posterior stabilized prosthesis to a nonlinked constrained, hinged or rotating hinge device is required [1, 2, 6–11]. In cases of gross segmental bone loss, modular prostheses or allograft-prosthesis composites are used for reconstruction [8, 12].

Modular knee prostheses with a rotating hinge articulation are used for reconstruction following tumor resections around the knee, complex primary knee arthroplasty, and revision total knee arthroplasties. Such devices provide a stable reconstruction of the knee when the intrinsic soft-tissue stability had been lost as a result of surgical intervention [13–15].

The latest generation of modular rotating hinge knee prostheses allows motion in three planes. The hinge axis allows flexion-extension motion, and the vertically oriented post-in-channel axis provides internal and external rotations [15]. Furthermore, the post-in-channel design allows distraction during flexion and extension, which is restricted by the restraint of the remaining soft tissues [15]. If distraction of a rotating hinge prosthesis occurs, the angular laxity depends on the design of the central rotational stem (length and taper) and the design of the tibial rotational cylinder [15].

Dislocation of rotating hinge prostheses due to implant's breakage or fatigue failures is occasionally observed in patients following total knee arthroplasty for tumoral indications and revisions total knee arthroplasty [2, 16–22].

Based on an earlier published biomechanical study, we tested six further implants of American and European manufacturers [15]. The aim of the biomechanical study was to establish the association between design of the central rotational stem and implant's stability. Therefore, the angular laxity of the peg with increasing amounts of distraction as well as the maximum amount of distraction before the stem's dislocation was determined. The hypothesis of the study was that implant designs with cylindrical shaped pegs are more stable than implant designs with conical shaped pegs concerning stability and maximum amount of distraction before dislocation.

## 2. Material and Methods

**Figure 1:**
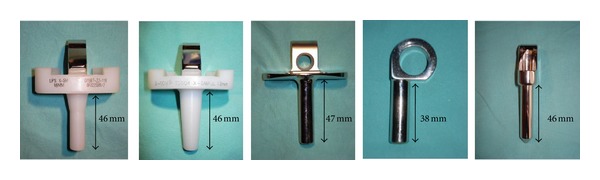
The length and the shape of the tested polyethylene inlays/central rotational stems of five rotating hinge knee prostheses used for the biomechanical analysis: LPS/M.B.T., S-ROM Noiles, GMRS, RT-Plus, and NexGen (12 mm polyethylene inlay; from left to right).

We performed a biomechanical analysis using a custom made biomechanical apparatus on a test bench. Therefore, the lengths and tapers of the peg of six different rotating hinge knee implants (Limb Preservation System—LPS/M.B.T. (DePuy, Warsaw, IN); S-ROM Noiles (DePuy), Global Modular Resection System—GMRS (Stryker, Mahwah, NJ), RT-Plus (Plus Orthopedics, Mödling, Austria), and NexGen (Zimmer, Kiel, Germany)) were determined with a standard calliper rule (Figure 1, Table 1). The Zimmer NexGen was tested twice, one time with the thinnest and one time with the thickest polyethylene inlay available because the length of the peg varies with the thickness of the polyethylene inlay.

Additionally, we intended to test the LINK Endo Model (Waldemar Link GmbH and Co.KG, Hamburg, Germany) and the GenuX (Implantcast GmbH, Buxtehude, Germany), but both implants were not suitable for the biomechanical analysis because of their mechanical antidislocation feature which prevents distraction at all.

The degree of tilting of the central rotational stem within the vertical post-in channel was measured in a theoretical setting by extending the distraction, as well as the maximum amount of distraction before the stem's dislocation. Therefore, three different methods of measurement were performed.

**Figure 2:**
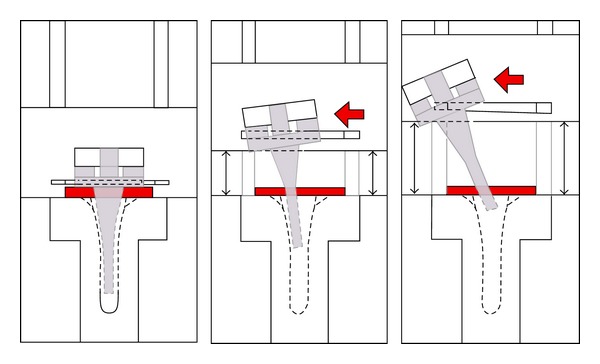
Graphic delineation of the custom made biomechanical apparatus describing its functional concept.

In the first method, a custom made biomechanical apparatus was used to imitate distraction, but physiological load was not applied. Nevertheless, the central rotational stem/polyethylene inlay was fixed to the proximal (femoral) base plate of the biomechanical apparatus (Figure 2), which was mobile to imitate the distraction (Figure 2). The hinged bearing inserts were fixed with a retainer to the proximal base plate with a mobile screw in a horizontal gliding slot to simulate the angulation. The tibial component was edged into the distal (tibial) part of the biomechanical apparatus with the rotational surface horizontally aligned. The vertical alignment of the components in the neutral position was verified with the centerline of a goniometer. Laterally directed, not standardized pressure, which was produced by each observer, was used to generate the angulation of the peg within the tibial rotational cylinder. This was done because we had no generic testing machine to apply physiological loading.

**Figure 3:**
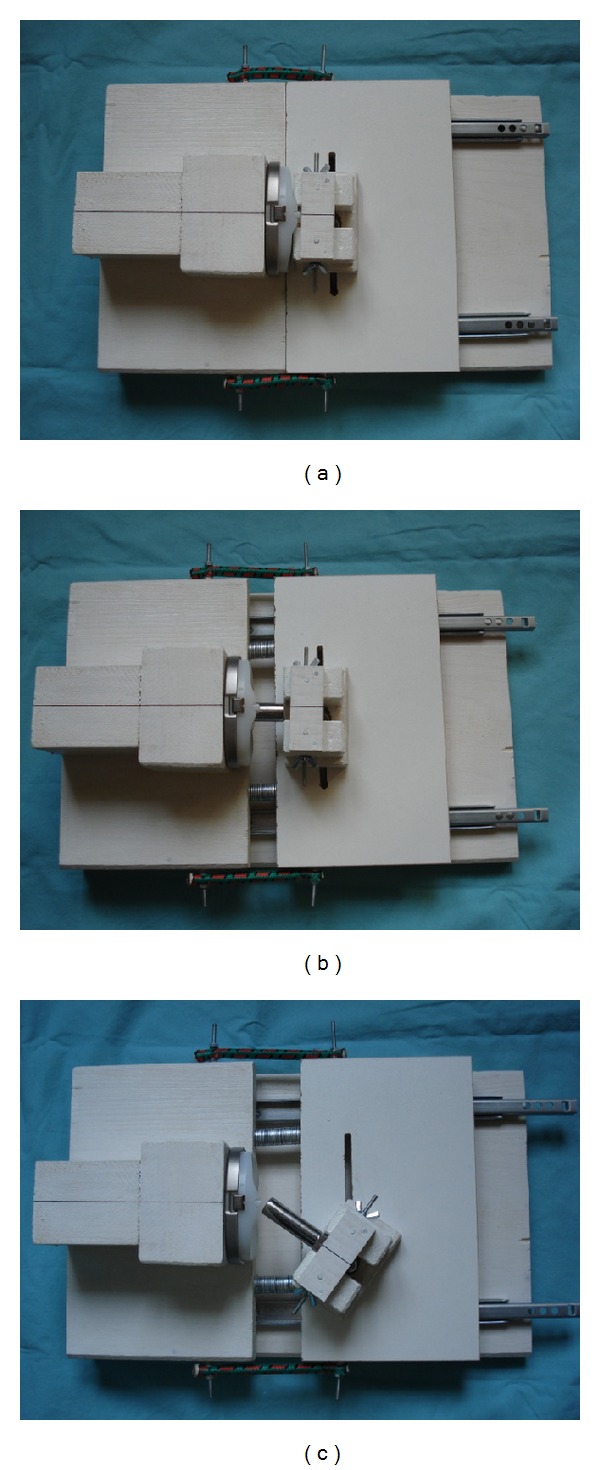
Photographs of the biomechanical apparatus testing the RT-Plus device. (a) RT-Plus device at 0 mm of distraction. (b) RT-Plus RHK at 20 mm of distraction. (c) Dislocated central rotational stem at 30 mm of distraction.

Displacement of the central rotational stem to each side was measured with a standard goniometer. Distraction was increased by 5 mm increments using 1 mm metal platelets, which were stacked, up to the increment that allowed dislocation (Figures 3(a)–3(c)). The point of dislocation was defined as the point at which any laterally directed force would cause the central rotational stem to jump off the tibial component (Figure 3(c)). After femorotibial disengagement, the measurements were repeated with 1 mm increments from the last 5 mm increment before the increment that allowed determining dislocation.

For the second approach, the fixation screw at the horizontal gliding slot was removed and the vertical post-in channel was filled with the metal platelets under the tip of the peg to simulate distraction as performed in the study of Ward et al. [15]. The purpose of this procedure was to evaluate if the horizontal gliding slot would influence the results of method 1. The remaining steps of measurement were repeated the same as in the first method.

In the third method, the measurements were visualized with sketches of the prosthesis components which were overlaid for each increment of distraction. The graphics were of original size.

**Figure 4:**
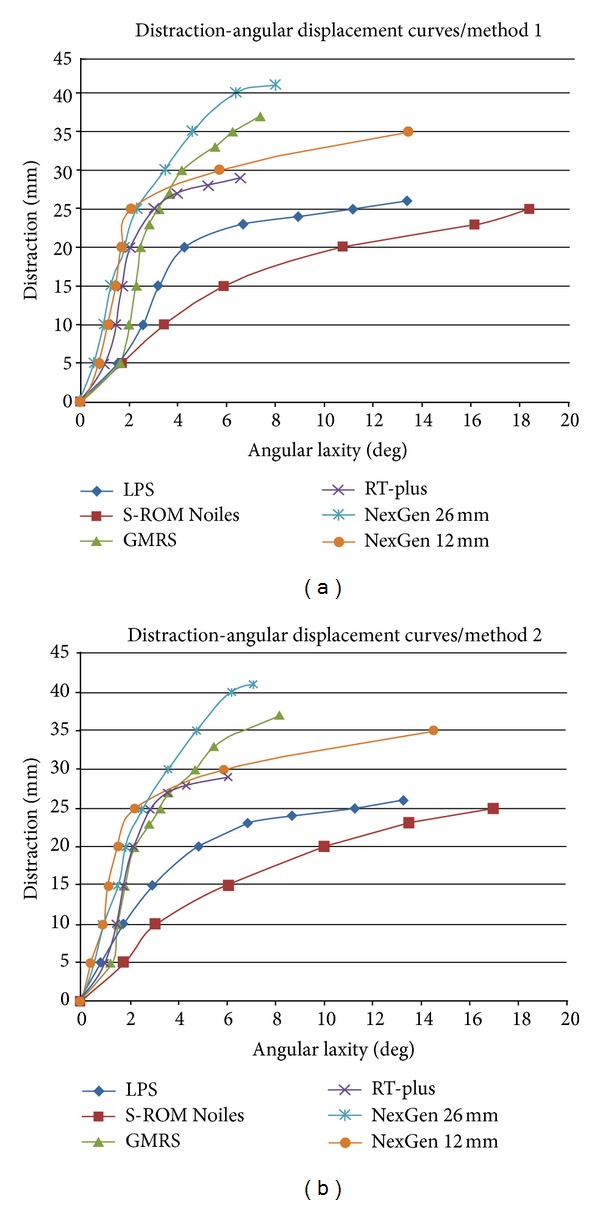
Distraction-angular displacement curves for the six tested knee designs, divided by methods 1 and 2. The final point of each curve (GMRS—37 mm, LPS/M.B.T.—26 mm, S-ROM Noiles—25 mm, RT-Plus—29 mm, NexGen with 12 mm PE—35 mm, and NexGen with 26 mm PE—41 mm) shows the last measureable angle before the implant's dislocation.

Three observers (Andreas Leithner, senior orthopedic surgeon, Mathias Glehr, resident, and Joerg Friesenbichler, resident) with different physiological strength performed three cycles of measurements with each implant and method. Mean distraction-angular displacement curves were generated for each device and method. The degrees of angular laxity were plotted at each level of the component's distraction. The slope of the curves reflects the extent to which the angular laxity increases, with increasing distraction. The end point of each curve demonstrates the last measureable angle before the implant's dislocation (Figure 4).

Multiple one-way analyses of variance (ANOVA) were performed at 10 mm, 15 mm, and 25 mm of distraction for methods 1 and 2 to determine if there were differences between the tested implants concerning stability against lateral directed forces (angular laxity). Furthermore, an intraclass-correlation coefficient (ICC) was calculated at 15 mm and 25 mm of distraction to determine the observer's reliability. Additionally, at the same increments, an interobserver correlation matrix was performed. We calculated observed power according to the magnitude of distraction-angular displacement at 10 mm, 15 mm, and 25 mm of distraction in both methods according to Hoenig and Heisey [23]. For statistical analysis the PASW Statistics 17.0 program (SPSS Inc., Chicago, IL, USA) was used, and a *P*  value < 0.05 was considered to be statistically significant.

## 3. Results

Using the thinnest polyethylene inlays available, the GMRS, NexGen, and RT-Plus implant designs, with truly cylindrical central rotational stems, were superior to the LPS/M.B.T. (part cylindrical and part conical) and the S-ROM Noiles (conical) implant designs, with regard to stability and maximum amount of distraction before dislocation (38 versus 36 versus 30 versus 27 versus 26 mm, resp.) (Table 2).

The GMRS, NexGen, and RT-Plus implant designs with long central rotational stems (47, 46, and 38 mm) required 38, 36, and 30 mm of distraction do dislocate. In comparison, the LPS/M.B.T. and S-ROM Noiles (both have a stem length of 46 mm) dislocated at 27 mm and 26 mm, respectively, of distraction (Table 2). The NexGen rotating hinge device with a 26 mm polyethylene inlay and a stem length of 60 mm dislocated at 42 mm of distraction, but the implant was quite unstable from 40 mm of distraction until dislocation. Despite a shorter central rotational stem, the RT-Plus device dislocated later than the LPS/M.B.T. and the S-ROM Noiles implants (30 versus 27 versus 26 mm).

The GMRS, RT-Plus, and NexGen (with a 26 mm PE-inlay) implants were the only designs with tilting angles less than 10° degrees at any given amount of distraction until dislocation. This circumstance could be verified with methods 1 and 2 but not with method 3. At 25 mm of distraction, the LPS/M.B.T. and S-ROM Noiles devices had an angular laxity of 11.2° and 18.4° degrees in case of method 1 and 11.3° and 17° degrees in case of method 2, while at the same increment the other tested implants showed tilting angles ranging from 2.1° to 3.2° degrees for method 1 and 2.3° to 3.3° degrees for method 2 (Table 3).

The moments, forces, and the elasticity on the devices, respectively, during the measurements with methods 1 and 2 could not be reproduced using the graphic delineations (method 3). Therefore, it was not possible to determine the correct angular laxity with this method.

The distraction-angular displacement curve of the S-ROM Noiles design showed an inferior increment (high laxity) when compared to the other implant designs, which showed steep rising slopes (lower laxity) (Figure 4). These findings confirmed that shorter and more tapered pegs have a greater angular laxity at any given amount of distraction.

The multiple one-way ANOVA analyses revealed statistical significant differences between the tested implants concerning stability (angular laxity) against lateral directed forces at 10 mm, 15 mm, and 25 mm of distraction for both methods of measurement (method 1: *P* < 0.001 at all tested increments; method 2: *P* = 0.031 at 10 mm, *P* < 0.001 at 15 mm, and 20 mm of distraction), and the magnitude of difference between angular laxity at 10 mm, 15 mm, and 20 mm in both methods was large enough that post hoc power analysis could show over 80% power [23].

Furthermore, the intraclass-correlation coefficient (ICC) revealed excellent results for both methods of measurement. At 15 mm of distraction, the interobserver reliability was 0.932 (*P* < 0.001) for the first method and 0.958 (*P* < 0.001) for the second one, indicating high precision. The ICC at 25 mm of distraction resulted 0.994 (*P* < 0.001) for method 1 and 0.993 (*P* < 0.001) for method 2. Determining the interobserver correlation at 15 mm and 25 mm of distraction also showed high precision although not using a standardized pulley system to generate angular laxity.

## 4. Discussion

Using a custom made biomechanical apparatus, the current study demonstrated that rotating hinge designs with long, cylindrical, and central rotational stems (GMRS, NexGen, and RT-Plus) were superior to implant designs with shorter and/or more tapered rotational stems in the theoretical setting. The LPS/M.B.T. and S-ROM Noiles implants require at least 26 mm and 27 mm, respectively, of distraction to dislocate. In contrast, the GMRS, the NexGen (with a 12 mm polyethylene inlay), and the RT-Plus devices with truly cylindrical, nontapered central rotational stems required 38 mm, 36 mm, and 30 mm of distraction to dislocate. The NexGen rotating hinge knee with a 26 mm polyethylene inlay dislocated at 42 mm of distraction. The implants with cylindrical, nontapered central rotational stems also had the lowest tilting angles at any given amount of distraction until dislocation, while the S-ROM Noiles implant showed the highest angular laxity throughout the biomechanical analysis (Table 3). One main limitation of the study was the manual pressure, which was used to generate the lateral tilting of the central rotational stem within the tibial rotational cylinder and was not standardized by using a loaded pulley system. Therefore, each observer influenced the measurements with his physical strength, similar to the study of Ward et al. Furthermore, in clinical setting the forces transmitted to the prostheses are influenced by many variables such as patient's age, height, weight, length of the lower extremities, muscle strength, cementation technique of the implant, and the patient's daily habits which were not minded in the current study. Nevertheless, determining the interitem correlation matrix revealed high interobserver agreement and could therefore diminish this bias.

Ward et al. [15] performed a biomechanical analysis testing the stability of seven rotating hinge knee designs. This study showed that devices with longer, nontapered pegs had provided the best results which could be proven in the current biomechanical study using another different rotating hinge designs. The authors concluded that short and great tapered pegs have greater instability and higher risk of dislocation at any given amount of distraction. On the contrary, the Endo-Model (Waldemar Link GmbH and Co.KG) has the shortest peg, and therefore this device would need the least distraction to dislocate. Nevertheless, this implant seems to be a safe design because of the mechanical antidislocation feature [15, 18].

Mild distraction of the knee is observed following endoprosthetic reconstruction. Investigations showed that instability, relative to soft-tissue compromise and ligamentous insufficiency, is most apparent to the patients in flexion when they lift the lower limb out of a seated position or leave the leg dangling [2, 18, 20, 24].

Kabo et al. [25] and Harrison Jr. et al. [10] tested the rotational stability of a rotating hinge device and demonstrated the importance of the soft tissues and the newly formed periprosthetic scar, to protect the prosthesis from excessive rotational stresses, especially when using devices without a rotational stop.

Medial and/or lateral instabilities following total knee arthroplasty are of concern. Ligamentous imbalance, component malalignment, or loosening and polyethylene wear are the most common reasons for this complication [26]. Depending on the severity of instability, several repair techniques and treatment options have been described. Nonoperative management includes knee immobilizer, braces, or orthoses [26]. Further opportunities to enhance the stability are advancement of the collateral ligaments, muscle, and/or tendon transfers. Additionally, collateral ligament insufficiency can be treated with soft-tissue reconstruction and/or with implants that provide inherent stability in the coronal plane [27].

Dislocation of rotating hinge knee prostheses is a rare complication. In the majority of cases, except for those of Ward et al. [20], Petrou et al. [22], and Joshi and Navarro-Quilis [2], dislocation occurred due to breakage of any prosthesis' component or fatigue of the tibial antidislocation device. All authors supposed excessive flexion gap instability and posterior dislocating forces causing the mechanical failure of the prostheses [2, 16–20]. C.-J. Wang and H. E. Wang [19] and Pacha-Vicente et al. [18] emphasized the importance of the ligamentous balance, especially the flexion gap, and suggested constrained prosthesis with an antidislocation device in cases of ligamentous insufficiency [18, 19]. Ward et al. [15, 20] recommended rotating hinge devices with a long (>5 cm), cylindrical peg or an effective mechanical antidislocation feature in case of severe articular compromise.

## 5. Conclusion

The results of the biomechanical analysis showed that the design of the peg plays a major role in the stability of a rotating hinge device. We conclude that rotating hinge prostheses with shorter and markedly tapered pegs have the highest angular laxity at any given amount of distraction, and they may become unstable under conditions of mild joint distraction, theoretically. Prosthetic designs with longer, cylindrical pegs might be useful in patients with severe articular compromise because the intrinsic design of such devices allows less tilting under mild joint distraction. Nevertheless, none of the implants allowed dislocation until at least 25 mm of distraction. Furthermore, a clinical evaluation is indicated to verify this recommendation. 

## Figures and Tables

**Table 1:** Manufacturer, stem length, taper, and height of the polyethylene inlay of six tested rotating hinge knee devices.

Manufacturer	Stem length (mm)	Stem taper (deg)	Polyethylene inlay (mm)
Stryker—GMRS	47	0	10
DePuy—LPS/M.B.T.	46	0	—
DePuy—S-ROM Noiles	46	5	—
PLUS Orthopedics—RT-Plus	38	0	8
Zimmer—NexGen	46	0	12
Zimmer—NexGen	60	0	26

**Table 2:** Results of the biomechanical analysis using the custom made biomechanical apparatus (Figures 3(a)–3(c)).

Manufacturer	Stem length (mm)	Stem taper (deg)	Polyethylene inlay (mm)	Minimum distraction to dislocate (mm)	AL at 25 mm of distraction (deg)/method 1	AL at 25 mm of distraction (deg)/method 2
Stryker—GMRS	47	0	10	38	3,2 (SD: 0,33)	3,3 (SD: 0,61)
DePuy—LPS/M.B.T.	46	0	—	27	11,2 (SD: 0,80)	11,3 (SD: 1,28)
DePuy—S-ROM Noiles	46	5	—	26	18,4 (SD: 1,73)	17,0 (SD: 1,13)
PLUS Orthopedics—RT-Plus	38	0	8	30	3,0 (SD: 0,90)	2,9 (SD: 0,90)
Zimmer—NexGen	46	0	12	36	2,1 (SD: 0,68)	2,3 (SD: 0,66)
Zimmer—NexGen	60	0	26	42	2,3 (SD: 0,32)	2,6 (SD: 0,44)

AL: angular laxity; SD: standard deviation.

**Table 3:** Angular laxity according to the amount of distraction, divided by method 1 and method 2.

Distraction (mm)	Angular laxity (deg)					
	LPS/M.B.T.	S-ROM Noiles	GMRS	RT-Plus	NexGen 12 mm	NexGen 26 mm
	Method 1					
5	1,6 (SD: 0,26)	1,8 (SD: 0,21)	1,6 (SD: 0,21)	1,0 (SD: 0,36)	0,8 (SD: 0,26)	0,6 (SD: 0,29)
10	2,6 (SD: 0,23)	3,4 (SD: 0,65)	2,0 (SD: 0,05)	1,5 (SD: 0,53)	1,1 (SD: 0,65)	1,0 (SD: 0,13)
15	3,2 (SD: 0,33)	5,9 (SD: 1,63)	2,3 (SD: 0,15)	1,7 (SD: 0,48)	1,5 (SD: 0,69)	1,3 (SD: 0,25)
20	4,3 (SD: 0,53)	10,7 (SD: 0,08)	2,5 (SD: 0,10)	2,1 (SD: 0,50)	1,7 (SD: 0,62)	1,8 (SD: 0,08)
25	11,2 (SD: 0,80)	18,4 (SD: 1,73)	3,2 (SD: 0,33)	3,0 (SD: 0,90)	2,1 (SD: 0,68)	2,3 (SD: 0,32)
30	—	—	4,2 (SD: 0,28)	—	5,7 (SD: 3,10)	3,5 (SD: 0,85)
35	—	—	6,3 (SD: 0,96)	—	13,4 (SD: 1,64)	4,6 (SD: 1,21)
40	—	—	—	—	—	6,4 (SD: 0,63)

	Method 2					
5	0,9 (SD: 0,56)	1,8 (SD: 0,80)	1,3 (SD: 0,25)	1,1 (SD: 0,50)	0,5 (SD: 0,18)	0,6 (SD: 0,13)
10	1,8 (SD: 0,93)	3,1 (SD: 1,23)	1,6 (SD: 0,17)	1,5 (SD: 0,75)	0,9 (SD: 0,32)	1,0 (SD: 0,20)
15	3,0 (SD: 0,74)	6,1 (SD: 0,67)	1,8 (SD: 0,22)	1,8 (SD: 0,85)	1,2 (SD: 0,52)	1,6 (SD: 0,61)
20	4,9 (SD: 0,56)	10,0 (SD: 1,50)	2,2 (SD: 0,25)	2,2 (SD: 0,82)	1,5 (SD: 0,61)	1,9 (SD: 0,56)
25	11,3 (SD: 1,28)	17,0 (SD: 1,13)	3,3 (SD: 0,61)	2,9 (SD: 0,90)	2,3 (SD: 0,66)	2,6 (SD: 0,44)
30	—	—	4,7 (SD: 1,08)	—	5,9 (SD: 0,88)	3,6 (SD: 0,23)
35	—	—	6,7 (SD: 1,27)	—	14,5 (SD: 0,45)	4,8 (SD: 1,00)
40	—	—	—	—	—	6,2 (SD: 1,54)

SD: standard deviation.
